# Clinical Significance of Adropin and Afamin in Evaluating Renal Function and Cardiovascular Health in the Presence of CKD-MBD Biomarkers in Chronic Kidney Disease

**DOI:** 10.3390/diagnostics13193158

**Published:** 2023-10-09

**Authors:** Rupinder Kaur, Pawan Krishan, Pratima Kumari, Tanveer Singh, Varinder Singh, Ravinder Singh, Sheikh F. Ahmad

**Affiliations:** 1 Chitkara College of Pharmacy, Chitkara University, Rajpura 140401, Punjab, India; rupinder@chitkara.edu.in (R.K.); pratima.kumari@chitkara.edu.in (P.K.); 2 Department of Pharmaceutical Sciences and Drug Research, Punjabi University, Patiala 147002, Punjab, India; pawankrishan@rediffmail.com; 3 Department of Neuroscience and Experimental Therapeutics, College of Medicine, Texas A&M Health Science Center, Bryan, TX 77807, USA; tanveersingh1988@gmail.com; 4 Department of Pharmaceutical Sciences and Technology, Maharaja Ranjit Singh Punjab Technical University, Bathinda 151001, Punjab, India; varinderjassal17@gmail.com; 5 Department of Pharmacology and Toxicology, College of Pharmacy, King Saud University, Riyadh 11451, Saudi Arabia; fashaikh@ksu.edu.sa

**Keywords:** CKD, CKD-MBD, adropin, afamin, cardiovascular, biomarker

## Abstract

Aim: The study aims to test the hypothesis that concentrations of adropin and afamin differ between patients in various stages of chronic kidney disease when compared with healthy controls. The study also investigates the association of the biomarkers (adropin and afamin) with CKD-MBD and traditional cardiovascular risk parameters in CKD patients. Methodology: The cross-sectional study includes the subjects divided into four groups comprising the control group (healthy volunteers = 50), CKD stages 1–2 patients (*n* = 50), CKD stages 3–4 patients (*n* = 50), CKD stage 5 patients (*n* = 50). Serum concentrations of adropin and afamin were determined using ELISA. Clinical variables (renal, lipid, and CKD-MBD parameters) were correlated to adropin and afamin concentrations. Results: Afamin concentration was found to be higher in group IV, followed by groups III and II when compared to the control group, i.e., (83.243 ± 1.46, 64.233 ± 0.99, and 28.948 ± 0.72 vs. 14.476 ± 0.5) mg/L (*p* < 0.001), and adropin concentration was found to be lower in group IV as compared to groups III, II, and I (200.342 ± 8.37 vs. 284.682 ± 9.89 vs. 413.208 ± 12.32 vs. 706.542 ± 11.32) pg/mL (*p* < 0.001), respectively. Pearson correlation analysis showed that afamin was positively correlated with traditional cardiovascular risk biomarkers, while adropin showed a negative correlation. Conclusions: Adropin and afamin may potentially serve as futuristic predictors for the deterioration of renal function and may be involved in the pathological mechanisms of CKD and its associated complications such as CKD-MBD and high lipid levels.

## 1. Introduction

Cardiovascular risk and its related complications are higher in patients with chronic kidney disease (CKD), which further causes mortality in CKD patients. The American Heart Association and National Kidney Foundation identify chronic kidney illness as a risk equivalent to coronary heart disease, an independent risk factor for cardiovascular disease [1]. Patients with renal failure are more likely to have cardiovascular disease due to the higher incidence of traditional cardiovascular risk factors, including diabetes and hypertension, but other factors, including inflammation, oxidative stress, endothelial dysfunction, dyslipidemia, anemia, malnutrition, and imbalanced mineral homeostasis, also play a major role [2]. Stages of atherothrombosis such as early cell adhesion, matrix and collagen degradation, endothelium damage, smooth muscle cell proliferation, enhanced platelet activity, ruptured plaque, thrombosis, and the development of vascular calcification are all promoted by the chronic inflammatory state in patients with CKD [3,4]. Dyslipidemia in CKD encircles the atherogenic profile expressed by the higher levels of triglycerides (TG), low levels of high-density lipoprotein (HDL), and changes in low-density lipoprotein (LDL). TG levels are increased in CKD [5]. Furthermore, impaired mineral ion metabolism leads to mineral bone disorder (MBD) in CKD, especially due to serum phosphorous, parathyroid hormone (PTH), Vitamin D, and calcium. In CKD-MBD, blood vessels in CKD begin to mineralize as the expressions of calcific inducers increase and the expressions of anticalcific proteins decrease. It increases cardiovascular risk through the pathological mechanism of vascular calcification. Vascular calcification is caused by the acceleration of mineral deposition in blood arteries due to CKD-MBD [6]. Vascular calcification causes blood calcium deposition in the blood vessels due to hyperparathyroidism in CKD. This further decreases the aortic and arterial resistance and leads to atrial stiffness, endothelial dysfunction, increasing arterial wave velocity in calcified arteries. This increased arterial wave velocity and dyslipidemia impact cardiovascular hemodynamics, leading to cardiovascular events such as hypertension, myocardial infarction, congestive heart failure, cardiac hypertrophy, etc., further contributing to life-threatening outcomes in CKD. The primary process in vascular calcification is the differentiation of vascular smooth muscle cells into osteoblast-like cells [7,8].

As cardiovascular complication is one of the predisposing factors for mortality in CKD, it is of utmost importance to evaluate the cardiac health in CKD at the earliest stages. Although traditional biomarkers of cardiac health, such as lipid profile, are the important predictors of progression to cardiac dysfunction, these estimations are not enough for the early prediction and monitoring of the disease development [2,9]. Moreover, CKD-MBD biomarkers also elevate the decline in renal function. This added the importance of checking the CKD-MBD biomarker levels in the CKD population [9]. Various secreted peptides maintain cardiovascular and metabolic homeostasis at the cellular level. Adropin is a peptide when administered to mice and rats; it tends to stimulate the signaling pathways in mammalian cell lines. Adropin regulates metabolic homeostasis in cardiac cells by mediating signaling pathways such as the G-coupled protein receptor 19 (GPR19)–mitogen-activated protein kinase (MAPK)–pyruvate dehydrogenase lipoamide kinase isozyme 4 (PDK4) pathway, VEGFR2-PI3K-Akt or VEGFR2-ERK1/2 pathways, and NB3/Notch signaling pathway. There are pieces of evidence that show that adropin can reduce inflammation by preventing the production of a variety of pro-inflammatory cytokines, including tumor necrosis factor alpha, C-reactive protein, and interleukin-6. Additionally, adropin may raise levels of HDL cholesterol while decreasing serum TG, total cholesterol, and LDL cholesterol and improving cardiac function and coronary blood flow [10,11].

It has also been hypothesized in the literature that adropin expressions in plasma regulate the endothelial function and the function of nitric oxide synthase. Impaired endothelial dysfunction contributes to the development of cardiovascular dysfunction [12,13]. Adropin, encoded by the energy-homeostasis-associated (ENHO) gene, is mainly demonstrated in tissues such as the liver, brain, heart, kidney, pancreas, coronary artery, and umbilical vein, and its expression is the highest in the brain [14]. Research studies have reported reduced expressions of adropin in metabolic syndrome, hyperlipidemia, obesity, and polycystic ovary syndrome, which are associated with inflammation. So, from here, it may be suggested that adropin deficiency triggers the inflammatory pathways [12,13]. Animal studies on diabetic mice have investigated the effective role of adropin in improving insulin resistance, and some data reported lower levels of adropin in patients with diabetes when compared with healthy volunteers. However, preclinical indicates the protective value of adropin in diabetic nephropathy [12,15]. To our knowledge, there have not been clinical studies considering the possible association of adropin in various stages of CKD patients to predict renal and cardiovascular health. So, the current study was planned to investigate the serum adropin concentrations in the CKD population.

On the contrary, afamin is the human plasma Vitamin E-binding glycoprotein of the albumin gene family primarily expressed in the liver, brain, kidneys, testes, and ovaries. There is not much information on the physiological roles of afamin. Lines of evidence state that afamin could have a role in neuroprotection, fertility, Vitamin E bioavailability, and bone metabolism. Afamin is strongly associated with the development of metabolic syndrome in animal studies (transgenic mice). Elevated levels of afamin in mice are positively correlated with the increase in body weight, total cholesterol, TG, and glucose levels that induce the state of hyperlipidemia [16]. Moreover, high urine afamin concentrations are also linked with glomerulonephritis [17]. However, an afamin assessment in the various stages of CKD patients has not been conducted. It is reasonable to assume a potential link with afamin since the CKD environment is linked with hyperlipidemia and chronic-grade inflammation. As very little is known about the pathological role of afamin in pathological conditions, we hypothesized the evaluation of the association of afamin with renal parameters and cardiovascular health biomarkers, including the lipid profile, blood pressure, and CKD-MBD biomarkers, in various stages of CKD.

Moreover, compared with healthy controls, adropin and afamin have not been studied together in CKD patients. The current study aimed to test the hypothesis of whether expressions of adropin and afamin change between patients in various stages of CKD with change in the renal parameters and CKD-MBD parameters to assess the renal function and cardiovascular health.

## 2. Material and Methods

### 2.1. Study Design and Patients

In this cross-sectional study, 150 CKD patients and 50 healthy subjects were recruited according to the inclusion/exclusion criteria, and written informed consent was obtained from all the study participants. CKD patients of either stage (1–5) were screened per the National Kidney Foundation CKD classification criteria by the physician based on the Glomerular filtration rate (GFR). Patients were included in the study from the tertiary care hospital in North India. Patients were excluded from the study if they had any history of stroke, malignancy, congenital heart disease, diabetes mellitus, or patients receiving estrogen or steroidal therapy. CKD patients (20–50 years) were then divided into four groups, Group II (CKD stage 1–2 patients), Group III (CKD stage 3–4 patients), and Group IV (CKD stage 5 patients who were on hemodialysis from the past one or more years), consisting of 50 patients in each group (*n* = 50). For comparison, 50 healthy individuals as a control group were included. The study protocol was approved by the Institutional Human Ethical Committee (IHEC) of Chitkara University, Punjab, and the study was conducted according to the principles of the Declaration of Helsinki and the Code of Good Clinical Practice (GCP).

### 2.2. Measurements of Clinical Variables/Biomarkers

To separate the serum, serum samples were collected and centrifuged for 10 min at approximately 2000–3000 rpm. The respective supernatants were collected in pyrogen-free and endotoxin-free disposable Eppendorf tubes and stored at −20 degree Celsius until analysis. The subject details and physical examination such as age, body weight, duration of dialysis (for ESRD patients), determination of systolic blood pressure (SBP), diastolic blood pressure (DBP), and the assessment of serum renal biomarkers (urea and creatinine), CKD-MBD biomarkers (calcium, phosphorous, urea, and Vitamin D), lipid biomarkers (LDL, TG, HDL, and cholesterol) of the recruited subjects were conducted during their routine clinical visits. Serum concentrations of adropin and afamin were analyzed using an enzyme-linked immunosorbent assay (ELISA, Wuhan Fine Biotech Co., LTD.) according to the manufacturer’s protocol. Blood samples for the adropin and afamin estimation were taken on the same day when other clinical variables were measured. 

### 2.3. Statistical Analysis

One-way analysis of variance (ANOVA) was used to compare the significant changes between all the groups in terms of clinical variables and biochemical parameters, followed by the Tukey’s post hoc test. All the data were expressed as Mean ± Standard Error Mean (SEM). The results were analyzed using Graph Pad Prism version 9. *p* ≤ 0.05 was considered as statistically significant. To find the association of serum levels of adropin and afamin with the CKD variables, CKD-MBD variables (calcium, phosphorous, and Vitamin D), and cardiovascular risk biomarkers (SBP, DBP, HDL, LDL, TG, and total cholesterol), Pearson correlation analysis was carried out as the data passed the normality test conducted with the Shapiro–Wilk test. In addition, the relationship between serum adropin, afamin, and all other biochemical variables was determined using multilinear regression analysis.

## 3. Results

### 3.1. Demographic and Biochemical Profile of the Study Participants

The current study was designed and aimed to assess the serum detection of the proteins (adropin and afamin) and MBD biomarkers (calcium, phosphorous, and Vitamin D) in the study, including the CKD patients with CKD stages 1–2, CKD stages 3–4, and CKD stage 5. Traditional risk biomarkers (SBP, DBP, TG, LDL, HDL, and total cholesterol) were also investigated in the study population. The demographic and biochemical profile of the included population is shown in Table 1.

### 3.2. Correlation Analysis

**Figure 1:**
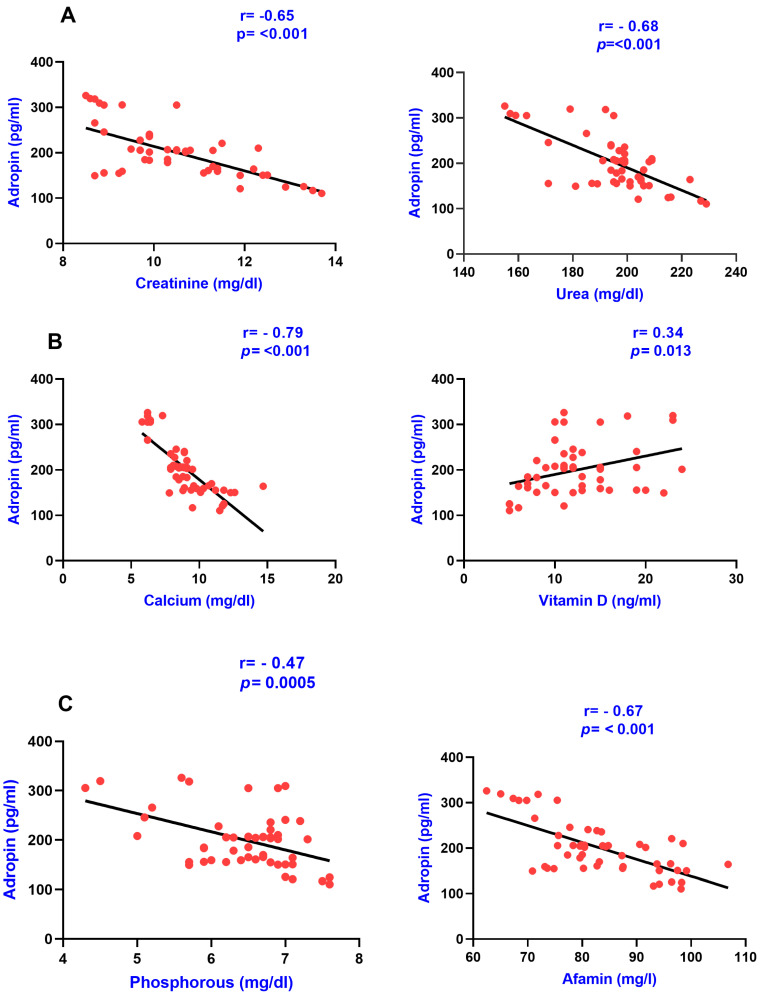
Pearson correlation analysis among serum adropin levels, renal parameters, CKD-MBD parameters, and afamin. The correlation is depicted between serum adropin levels and creatinine as well as with urea in panel (**A**), between adropin levels and calcium as well as with Vitamin D in panel (**B**), and between adropin levels and phosphorous as well as with afamin in panel (**C**).

**Figure 2:**
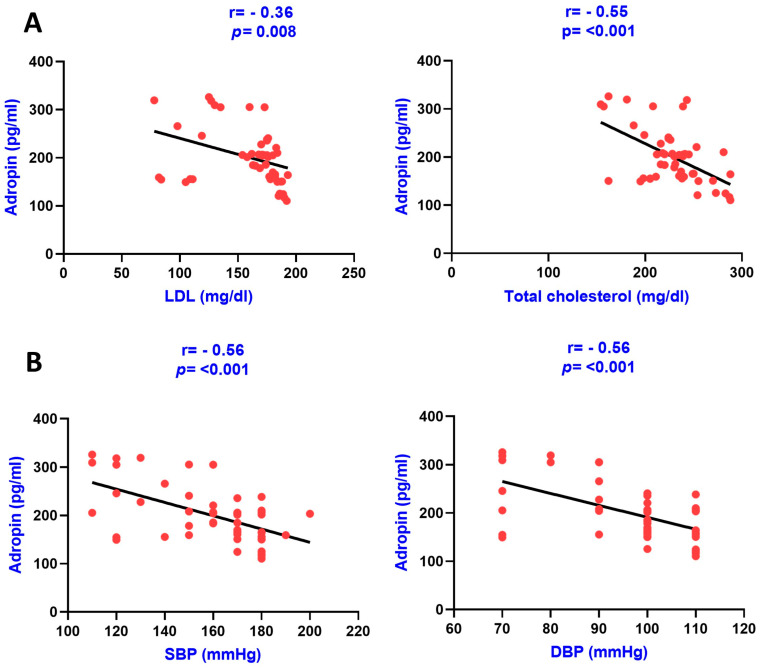
Pearson correlation analysis among serum adropin levels, lipid parameters, and blood pressure. The correlation is depicted between serum adropin levels and LDL as well as with total cholesterol in panel (**A**) and between adropin levels and systolic blood pressure as well as with diastolic blood pressure in panel (**B**).

The association of serum adropin with various clinical variables, including renal parameters, lipid parameters, CKD-MBD parameters (calcium, phosphorous, and Vitamin D), and protein—namely afamin—were carried out using the Pearson correlation analysis in group IV, group III, and in group II as presented in Table 2. Adropin was found to be negatively and significantly correlated with afamin, creatinine, urea, phosphorous, calcium, LDL, TG, blood pressure, and total cholesterol, and a positive association was found with Vitamin D in group IV. The correlation graphs of adropin with various clinical variables in group IV are shown in the figures below (Figure 1 and Figure 2).

**Figure 3:**
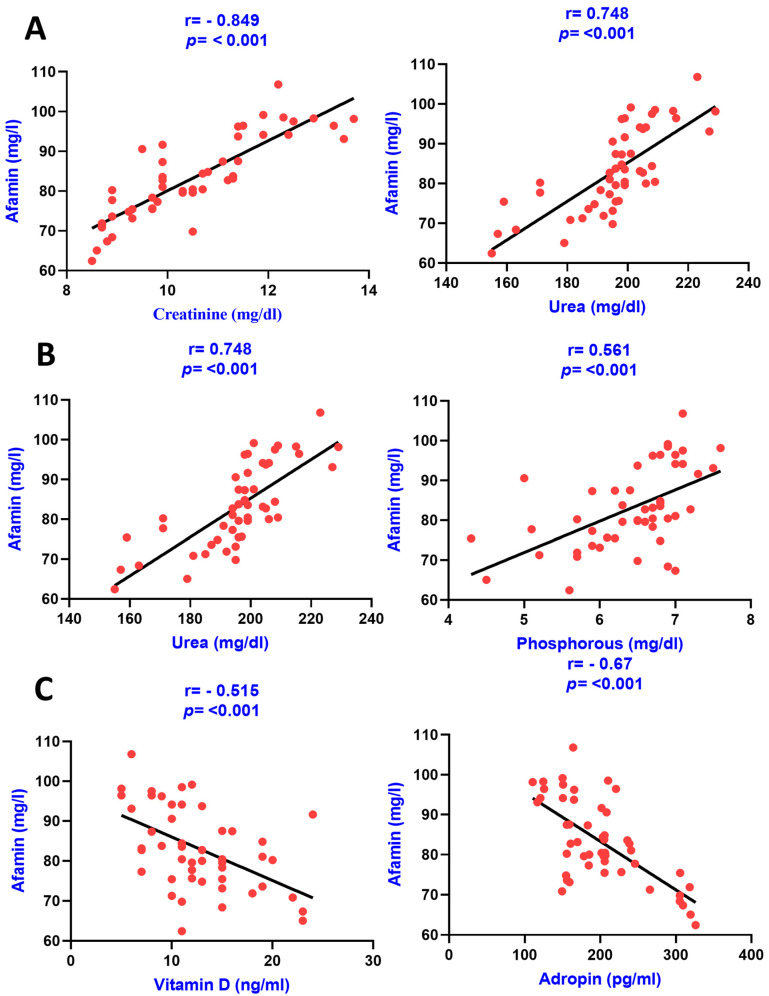
Pearson correlation analysis among serum afamin levels, renal parameters, CKD-MBD parameters, and adropin. The correlation is depicted between serum afamin levels and creatinine as well as urea in panel (**A**), between afamin levels and calcium as well as with phosphorous in panel (**B**), and between afamin levels and Vitamin D as well as with adropin in panel (**C**).

**Figure 4:**
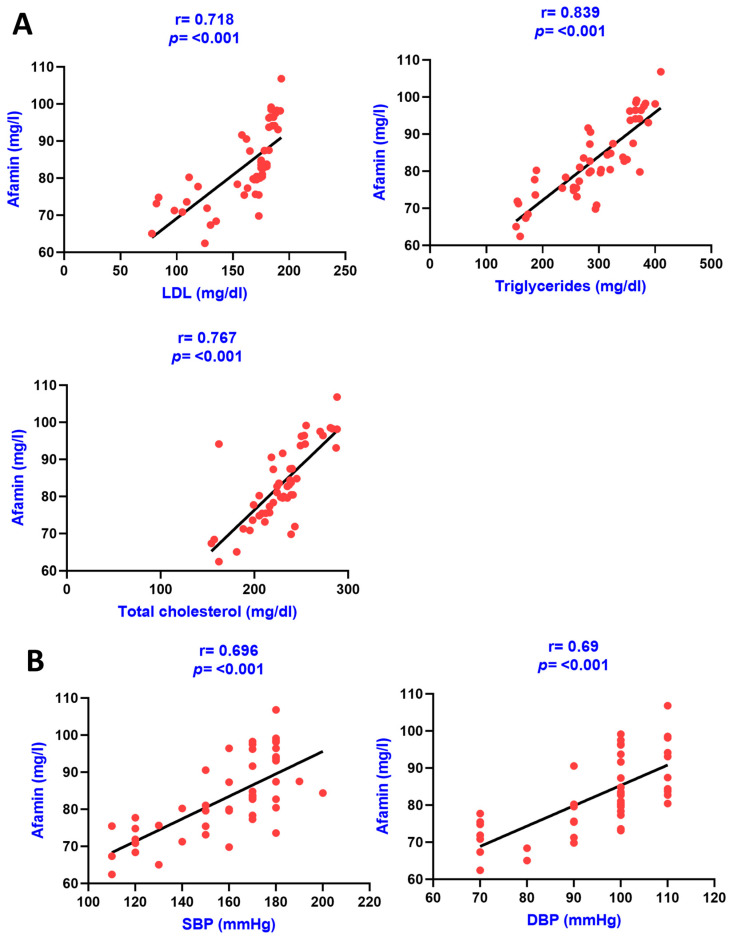
Pearson correlation analysis among serum afamin levels, lipid profile, and blood pressure. The correlation is depicted between serum afamin levels and LDL as well as with triglycerides, and total cholesterol in panel (**A**) and between afamin levels and systolic blood pressure as well as with diastolic pressure in panel (**B**).

Similarly, the association of serum afamin with various clinical variables in CKD patients was also carried out using the Pearson correlation analysis as results are shown in Table 3. The correlation graphs of afamin with various clinical variables in group IV are shown in the figures below (Figure 3 and Figure 4).

Furthermore, multiple linear regression analysis presented in Table 4, depicted that creatinine in groups IV, III, and II (*p* = 0.04, 0.0002, and 0.02) were significantly correlated with afamin depicting the role of afamin in renal dysfunctioning. Adropin and calcium were also significantly correlated with afamin in group IV patients followed by Vitamin D in group II, LDL in group III, and total cholesterol in group II patients. Similarly, in multiple linear regression analysis presented in Table 5, creatinine in groups III and II (*p* = 0.0005 and 0.0001) were significantly correlated with adropin, followed by afamin in group IV; calcium in groups II, III, and IV; LDL and TG in group IV; and Vitamin D in groups II and III.

## 4. Discussion

CKD-MBD results from mineral and metabolic abnormalities and is the major pathophysiological link for cardiovascular disease in CKD and ESRD patients [5,7,8,18,19,20]. Moreover, increase in the CKD-MBD biomarkers leads to further CKD progression [9,21].

Vitamin D is an important parameter that should also be monitored to check the calcium levels in the blood, as Vitamin D deficiency is associated with mineral and bone disorders leading to vascular calcification in CKD patients. Vitamin D has a protective effect on vascular calcification in CKD via various mechanisms such as reduction in cholesterol and foam cell inhibition and cholesterol efflux in the macrophages and modulating vascular regeneration, modulation of the calciprotein particles, and matrix vesicles [22]. Our study also supported the previous findings of increased calcium and phosphorous levels in CKD compared to healthy controls and decreased levels of Vitamin D in CKD patients. However, to our knowledge, no study demonstrates the comparison and correlation of traditional cardiovascular risk biomarkers (lipids), non-traditional cardiovascular risk biomarkers such as CKD-MBD parameters, and novel biomarkers (adropin and afamin) in various stages of CKD. CKD is linked with high TG, low HDL, and altered lipoprotein composition. These altered lipid parameters contribute to CVD, especially in ESRD patients on dialysis. Thus, managing these parameters is crucial to potentially reduce the cardiovascular risk in CKD patients [23,24].

Adropin and afamin, the newly discovered peptides, correlate with inflammation and its associated conditions, such as hyperlipidemia and metabolic syndrome [25,26,27].

In the present study, patients undergoing hemodialysis in group IV had significantly lower levels of serum adropin, followed by group III (CKD 3 and 4) and group II (CKD 1 and 2) patients as compared to group I (healthy controls). In the present study, the normal concentration of adropin was found to be (706.542 ± 11.32) pg/mL in the healthy group, which was found to be lower than the findings in CKD patients such as 413.208 ± 12.32 in CKD stage 1 and 2 patients, 284.682 ± 9.89 in CKD stage 3 and 4 patients, and 200.342 ± 8.37 in CKD stage 5 patients. Lower levels of adropin were also supported by the previous findings in metabolic syndrome patients [13]. Decreased levels of adropin were supported by various studies in different disease populations. However, our study describes the decreased levels of adropin in CKD patients with the highest concentrations in ESRD patients. Furthermore, adropin stimulates the nitric oxide production, through which it relates with vascular protection. Increased total cholesterol, TG, and LDL levels lead to several disorders, including coronary artery disease, myocardial infarction, etc. In an animal model of hyperlipidemic rats, the study was conducted to check the influence of daily intraperitoneal administration of adropin for ten days on plasma glucose and lipid profiles. It has been observed that body weight and serum concentration of lipids (total cholesterol, LDL, and TG) were decreased, while the concentration of HDL was increased. However, the results were not statistically significant [28]. Moreover, decreased levels of adropin were observed in metabolic syndrome patients when compared with obese subjects [13].

In the current study, lower levels of serum adropin were inversely correlated with cardiovascular health biomarkers such as total cholesterol, TG, LDL, SBP, DBP, phosphorous, and calcium. At the same time, no correlation was observed with HDL in group IV. The positive association of adropin with Vitamin D was statistically significant. In group III and group II, all the parameters were negatively correlated, and Vitamin D was positively correlated, which was in line with previous findings of K. Ganesh et al., which stated that increased adiposity and hyperlipidemia are found to be correlated with decreased levels of adropin in adropin knockout mice [29]. Additionally, Ghoshal et al., in their study, also supported the inverse association between plasma adropin concentrations and LDL-C [12]. Our study supports the negative correlation of adropin with lipids and blood pressure, so this may be hypothesized that this connection could be one of the links between CKD and cardiovascular health. However, high creatinine is strongly associated with adropin, depicting that adropin plays a vital role in renal function.

A research study suggested a relationship between decreased adropin concentrations and increased obesity [12,30]. Serum adropin levels were significantly decreased in type 2 diabetic patients with metabolic dysfunction-associated fatty liver disease in a study by Li N et al. [31]. Moreover, a research study mentioned that intraperitoneal administration of adropin to hyperlipidemic rats for ten days effectively lowered serum TG levels, total cholesterol, LDL-C, and alkaline phosphatase [30]. The current study also supports that lowered adropin concentrations might influence the lipids level, which is crucial for cardiovascular health. In the present study, the correlation analysis in CKD patients showed that a decrease in serum adropin levels was negatively correlated with CKD-MBD biomarkers such as phosphorus and calcium while positively correlated with Vitamin D. In another clinical study, serum concentrations of adropin were found to be lowered in atrial fibrillation patients. In the study, authors suggested that adropin may protect against the development of atrial fibrillation by inhibiting inflammation, as inflammation is the major triggering factor of cardiovascular diseases [32].

In the current study, serum afamin concentrations were increased in ESRD patients, followed by CKD III and IV as well as CKD I and II patients compared to the control group. The serum levels of afamin were lower in the healthy group compared to CKD patients. The strongest positive and significant association of adropin and negative association of afamin with renal parameters in the current study suggest that adropin and afamin may possibly play a role in the CKD/ESRD pathophysiology. Additionally, increased serum afamin levels positively correlated with LDL, cholesterol, TG, creatinine, urea, blood pressure, and calcium phosphorous and negatively correlated with Vitamin D in group IV. However, the correlation of afamin with phosphorous in group III and association with blood pressure and calcium in group II showed a weak association with non-significant results. These results are in line with the previous findings in metabolic syndrome patients. In the study, increased afamin levels were linked with metabolic syndrome markers such as high blood glucose, dyslipidemia, obesity, high blood pressure, type 2 diabetes mellitus, and pre-eclampsia [16,33]. A study found that urine afamin (uAFM) and afamin/creatinine ratio (AfCR) increased in patients with primary membranous and IgA nephropathy. Also, uAFM and AfCR positively correlate with urine albumin and albumin/creatinine ratio [17]. In the current study, multilinear regression shows the significant results of creatinine with afamin as compared to other parameters, depicting that afamin plays a pivotal role in renal dysfunction. However, other parameters such as lipid and CKD-MBD biomarkers could not be ignored as their increase in the CKD population may influence cardiovascular health. Hence, serum afamin and investigations on other parameters such as serum phosphorous, Vitamin D, and serum calcium may act as diagnostic parameters to diagnose and check the progression of renal dysfunction in CKD patients and may act as a therapeutic target.

In addition, various preclinical and clinical studies supported the protective role of adropin in cardiovascular risk. Adropin regulates metabolic homeostasis in cardiac cells through GPR19-MAPK-PDK4 pathway [34,35]. A study conducted on rats has shown that adropin promotes the proliferation of preadipocytes and inhibits their differentiation into mature adipocytes via mediating ERK1/2 and AKT, thus reducing lipid accumulation and adipogenic gene expressions [36]. Adropin may exhibit antioxidative effect. Studies have demonstrated that adropin deficiency in rats is associated with increased oxidative stress, leading to endothelial dysfunction in the brain [37]. Adropin upregulates the expression of endothelial nitric oxide synthase by activating VEGFR2-PI3K-Akt or VEGFR2-ERK1/2 signal transduction pathways in both in vitro and in vivo settings. Consequently, adropin may protect endothelial cells from nephropathy by promoting anti-inflammatory factors and NO production while alleviating oxidative stress, as CKD is also an inflammatory state. Adropin’s metabolic effects appear to offer protection to the renal tubular apparatus [34]. Low levels of adropin may indicate a maladaptive shift in energy homeostasis, characterized by overexpression of pro-inflammatory genes and excessive production of inflammatory cytokines, particularly in the context to oxidative stress and mitochondrial dysfunction [35,38]. In a clinical context, these findings indicate that adropin exerts tissue-protective capabilities through various mechanisms and that low levels of this circulating peptide may serve as an additional marker of renal dysfunction and cardiovascular health. Our study also supports the decreased levels of adropin in the CKD population, and renal function declines as the adropin level decreases. The close relationship of adropin with endothelial dysfunction and microvascular dysregulation may explain its role in predicting CKD, as these factors have been implicated as triggers of kidney injury leading to CKD. Furthermore, increased afamin levels have been linked with the development of the metabolic syndrome such as high blood glucose, dyslipidemia, obesity, and type 2 diabetes mellitus. Elevated levels of afamin in mice are positively correlated with the increase in body weight, cholesterol, triglycerides, and glucose levels that induce the state of hyperlipidemia [16,17,27,33]. In addition, Afa/Cre was elevated in T2DM patients who subsequently developed diabetic nephropathy [39]. So, the present study also supports the finding of the previous studies suggesting adropin and afamin may act as an add-on to the older laboratory biomarkers for assessing renal dysfunction and cardiovascular health in CKD patients.

CKD patients with mineral bone abnormalities seek more attention in managing mineral metabolism and traditional risk factors to prevent harmful effects on the vascular part and to diagnose renal health with the effective use of novel biomarkers.

### Limitations

The current study also has some limitations. The study does not include the ESRD patients on peritoneal dialysis compared to the hemodialysis patients. Moreover, the lack of vascular calcification assessment via Doppler ultrasound is also the limiting factor, as vascular calcification is the major pathological link leading to cardiovascular events in CKD. Vascular calcification can predict cardiovascular health in CKD patients. More studies on different disease populations need to be conducted to understand the mechanisms or pathways by which adropin maintains cardiac cell homeostasis. The nutritional status of the patients and use of several medications by CKD patients that might lead to any side effects or interactions have not been taken into account and could affect our results. Therefore, to identify a causal relationship between these biomarkers, genetic and multicenter studies on large cohort of patients are necessary to further specify and validate the findings of the current study and to confirm the prediction accuracy of adropin and afamin in the CKD population, along with other clinical factors and diagnostic techniques. There is a pressing need to further establish the physiological and pathological connection of adropin and afamin with renal and cardiovascular health in CKD population.

## 5. Conclusions

The findings from the present study suggest that a decrease in adropin and rise in afamin may potentially serve as futuristic predictors for the deterioration of renal function and may be involved in the pathological mechanisms of CKD and its associated complications such as CKD-MBD and high lipid levels. High lipid levels, blood pressure, and CKD-MBD biomarkers such as phosphorous, calcium, and Vitamin D could play an important role in predicting cardiovascular health in CKD. So, measuring the afamin and adropin concentrations along with CKD-MBD parameters may play an important role in the management of renal and cardiovascular health. Adropin and afamin may have a significant independent potential role as novel biomarkers to predict the futuristic development of renal dysfunction and cardiovascular complications, which may further have the potential to act as a therapeutic target in future. However, further genetic and multicenter studies on large sample sizes are necessary to specify further and validate the current study’s findings.

## 6. Future Directions

Adropin and afamin mediate metabolic homeostasis in various tissues and cells, including brain tissue, cardiomyocytes, vascular endothelial cells, fibroblasts, etc. According to several studies, adropin regulates various physiological processes such as energy metabolism, insulin sensitivity, and cardiovascular function. The molecular, biochemical, and structural aspects of adropin and afamin biology, physiology, mechanism of action still need to be investigated. More research is required to understand the signaling processes by which adropin and afamin function, particularly in various disease populations. Identifying these mechanisms in the vasculature and their subsequent signaling processes will make it easier to use adropin and afamin biology therapeutically to treat cardiometabolic and renal illnesses. Moreover, clinical research (diagnostic tools/biomarkers and therapeutic target) is continuously evolving. Therefore, the current study provides the future perspective of adropin and afamin potential to assess renal dysfunction.

## Figures and Tables

**Table 1:** Demographic and biochemical characteristics of recruited subjects.

Clinical Variables	Group I (Healthy Control) (*n* = 50)	Group II (CKD Stages 1–2 Patients) (*n* = 50)	Group III (CKD Stages 3–4 Patients) (*n* = 50)	Group IV (CKD Stages 5 Patients) (*n* = 50)
Age (years)	40.12 ± 0.26	40.23 ± 1.39	46.46 ± 1.98	48.18 ± 1.45
Gender (F/M)	28/22	19/31	25/25 *#	26/24 *#
SBP (mmHg)	120.9 ± 6.47	149.4 ± 3.08 *	154.8 ± 3.24 *	159.2 ± 3.36 *
DBP (mmHg)	81 ± 3.41	90.2 ± 1.57 *	93.8 ± 1.63 *	96.2 ± 1.84 *
Creatinine (mg/dL)	0.88 ± 0.01	1.86 ± 0.03 *	4.84 ± 0.18 *#	10.50 ± 0.19 *#†
Urea (mg/dL)	24.3 ± 3.2	91.12 ± 1.82 *	121.7 ± 3.44 *#	198.04 ± 1.69 *#†
Calcium (mg/dL)	6.48 ± 1.21	6.94 ± 0.11	7.52 ± 0.16 *	8.98 ± 0.24 *#†
Phosphorous (mg/dL)	4.11 ± 0.23	4.34 ± 0.16	5.59 ± 0.09 *#	6.44 ± 0.10 *#†
Vitamin D (ng/mL)	25.97 ± 1.99	20.58 ± 1.33 *	15.98 ± 0.80 *	12.54 ± 0.69 *#
Total cholesterol (mg/dL)	160.3 ± 4.56	181.46 ± 8.02	224.4 ± 7.81 *#	228.4 ± 4.66 *#
HDL (mg/dL)	56.7 ± 0.39	54.42 ± 0.56 *#	45.94 ± 0.31 *#	42.08 ± 0.81 *#†
LDL (mg/dL)	90.34 ± 4.18	114.34 ± 4.82 *	150.5 ± 4.25 *#	160.16 ± 4.50 *#
TG (mg/dL)	181.5 ± 14.12	192.93 ± 10.78	282.08 ± 11.06 *	293.54 ± 10.41 *#
Adropin (pg/mL)	706.542 ± 11.32	413.208 ± 12.32 *	284.682 ± 9.89 *#	200.342 ± 8.37 *#†
Afamin (mg/L)	14.476 ± 0.5	28.948 ± 0.72	64.233 ± 0.99	83.243 ± 1.46 *#

SBP: systolic blood pressure; DBP: diastolic blood pressure; HDL: high-density lipoproteins; LDL: low-density lipoproteins; and TG: triglycerides. Data are expressed as Mean ± SEM. *p* < 0.05 was considered statistically significant. * *p* < 0.05 vs. healthy control, # *p* < 0.05 vs. patients with CKD 1–2 (GP-I), † *p* < 0.05 vs. patients with CKD 3–4 (GP-II). One-way ANOVA followed by Tukey’s post hoc test was used to compare the groups.

**Table 2:** The Pearson correlation matrix of various clinical variables with adropin in groups IV, III, and II.

Adropin (pg/mL)	Group IV		Group III		Group II	
	r	*p*-Value	r	*p*-Value	r	*p*-Value
Afamin (mg/L)	−0.6702	<0.001	−0.751	<0.001	−0.505	<0.0002
Creatinine (mg/dL)	−0.6556	<0.001	−0.807	<0.001	−0.838	<0.001
Urea (mg/dL)	−0.6886	<0.001	−0.787	0.0008	−0.811	<0.001
Calcium (mg/dL)	−0.7964	<0.001	−0.7408	<0.001	−0.694	0.001
Phosphorous (mg/dL)	−0.4737	0.0005	−0.4598	0.0008	−0.469	0.0006
Vitamin D (ng/mL)	0.3464	0.0137	0.704	<0.001	0.558	<0.001
LDL (mg/dL)	−0.3679	0.0086	−0.826	<0.001	−0.606	<0.001
TG (mg/dL)	−0.7087	<0.001	−0.733	<0.001	−0.750	<0.001
Total cholesterol (mg/dL)	−0.5512	<0.001	−0.753	<0.001	−0.538	<0.001
HDL (mg/dL)	0.0316	0.8274	−0.0728	<0.001	−0.04	0.762
SBP (mmHg)	−0.5693	<0.001	−0.665	<0.001	−0.4357	0.002
DBP (mmHg)	−0.5615	<0.001	−0.751	<0.001	−0.3847	0.005

SBP: systolic blood pressure; DBP: diastolic blood pressure; HDL: high-density lipoproteins; LDL: low-density lipoproteins; and TG: triglycerides.

**Table 3:** Pearson correlation analysis of various clinical variables with afamin in groups IV, III, and II.

Afamin (mg/L)	Group IV		Group III		Group II	
	r	*p*	r	*p*	r	*p*
Adropin (pg/mL)	−0.670	<0.001	−0.7643	<0.001	−0.505	<0.002
Creatinine (mg/dL)	0.849	<0.001	0.916	<0.001	0.664	<0.001
Urea (mg/dL)	0.748	<0.001	0.8873	<0.001	0.545	<0.001
Calcium (mg/dL)	0.777	<0.001	0.5511	<0.001	0.1838	0.2012
Phosphorous (mg/dL)	0.561	<0.001	0.2186	0.12	0.329	0.020
Vitamin D (ng/mL)	−0.515	<0.001	−0.663	<0.001	−0.395	0.005
LDL (mg/dL)	0.718	<0.001	0.842	<0.001	0.556	<0.001
TG (mg/dL)	0.839	<0.001	0.790	<0.001	0.641	<0.001
Total cholesterol (mg/dL)	0.767	<0.001	0.857	<0.001	0.662	<0.001
SBP	0.694	<0.001	0.833	<0.001	0.3149	0.259
DBP	0.690	<0.001	0.767	<0.001	0.2045	0.154

SBP: systolic blood pressure; DBP: diastolic blood pressure; LDL: low-density lipoproteins; and TG: triglycerides.

**Table 4:** Multiple linear regression analysis between afamin and various biochemical parameters in CKD patients.

Afamin (mg/L)	Group IV		Group III		Group II	
	R^2^	*p* Value	R^2^	*p* Value	R^2^	*p* Value
Adropin (pg/mL)	0.74	0.05		--------		-------
Calcium (mg/dL)	0.71	0.0003		--------		-------
Phosphorous (mg/dL)		-------		--------		-------
Vitamin D (ng/mL)		-------			0.71	0.05
LDL (mg/dL)	0.75	0.36	0.94	0.05		
TG (mg/dL)		-------		--------		-------
TC (mg/dL)		-------			0.52	0.01
SBP (mmHg)		-------		--------		-------
DBP (mmHg)		-------		--------		-------
Creatinine (mg/dL)	0.88	0.04	0.95	0.0002	0.88	0.02

**Table 5:** Multiple linear regression analysis between adropin and various biochemical parameters in CKD patients.

Adropin (pg/mL)	Group IV		Group III		Group II	
	R^2^	*p* Value	R^2^	*p* Value	R^2^	*p* Value
Afamin (mg/L)	0.74	0.05				
Calcium (mg/dL)	0.66	<0.0001	0.52	0.02	0.36	0.0001
Phosphorous (mg/dL)		-------		--------		-------
Vitamin D (ng/mL)	0.49	0.76	0.62	0.05	0.61	0.05
LDL (mg/dL)	0.72	0.04		--------		-------
TG (mg/dL)	0.85	0.001		--------		-------
TC (mg/dL)		-------		--------		-------
SBP (mmHg)		-------		--------		-------
DBP (mmHg)		-------		--------		-------
Creatinine (mg/dL)	0.88	0.5	0.91	0.0005	0.86	0.0001

## Data Availability

The data presented in this study are available within the article.
